# Development of Emotional Skills in Adolescents to Prevent Cyberbullying and Improve Subjective Well-Being

**DOI:** 10.3389/fpsyg.2018.02050

**Published:** 2018-10-26

**Authors:** Konstanze Schoeps, Lidón Villanueva, Vicente Javier Prado-Gascó, Inmaculada Montoya-Castilla

**Affiliations:** ^1^Department of Personality, Assessment and Psychological Treatment, Universitat de València, Valencia, Spain; ^2^Developmental Psychology, Universitat Jaume I, Castelló de la Plana, Spain; ^3^Department of Social Psychology, Universitat de València, Valencia, Spain

**Keywords:** intervention program, emotional education, peer-to-peer coexistence, cyberbullying, life satisfaction, well-being, adolescents

## Abstract

Bullying behavior alters the way in which students coexist together in the classroom and negatively affects adolescents’ well-being. Research highlights the importance of emotional skills in promoting positive youth development and optimal social functioning. Therefore, education in these skills is a potential target for interventions aimed at reducing cyberbullying and promoting satisfaction with life during adolescence. This study analyzes the impact of an emotion education program in adolescents to promote classroom coexistence and well-being. The sample comprised 148 students from 7th and 8th grade of secondary school aged between 12 and 15 years (*M*_age_ = 12.63, *SD*_age_ = 0.74; 57% girls). A quasi-experimental design with longitudinal data collection was used in this study with randomized classroom assignment to the experimental group and the control group. The intervention program was based on the emotional intelligence model of Mayer and Salovey (1997). Its objective was to develop adolescents’ emotional skills to improve the quality of interpersonal relationships and reduce conflicts between peers, positively influencing coexistence and well-being. The intervention took place in eleven sessions during school hours over a period of 3 months. Participants completed the emotional competence questionnaire, the cyberbullying scale and the life satisfaction scale before (T1), immediately after (T2), and 6 months after the intervention (T3). The results showed that the intervention program reduced victimization and assault via mobile phones and the Internet in T2 and T3. In the follow-up (T3), the intervention group had enhanced emotional perception and regulation skills and reported an increase in life satisfaction in comparison to the control group. Our findings suggest that implementing classroom intervention programs to develop students’ emotional competencies could be beneficial for their subjective well-being and peer coexistence.

## Introduction

Learning to live together is a necessary and fundamental objective for the integral development of a student’s personality (Olweus and Limber, 2010). Research has shown that school violence (bullying and cyberbullying) alters peaceful coexistence (Smith, 2013). The benefits of emotional competencies on classroom coexistence and their positive impact on bullying prevention and adolescents’ well-being have been studied over the last few decades (e.g., Schokman et al., 2014; Elipe et al., 2015; Marikutty and Joseph, 2016). To address this problem, intervention programs have been designed to prevent traditional bullying and cyberbullying behavior (Ttofi and Farrington, 2010; Zych et al., 2015). However, bullying prevention programs that focus on social-emotional development and include aspects of adolescents’ subjective well-being are rare (Durlak et al., 2011). This study attempts to fill these gaps by evaluating not only the effectiveness of a social-emotional education program to promote coexistence in the classroom in relation to cyberbullying behaviors but also the impact on adolescents’ well-being over 6 months.

### Coexistence and Well-Being in the Classroom

School coexistence requires students to learn to relate to and interact with the people with whom they share daily time (school hours) and space (classroom): students and teachers. In the case of student–student relationships, peer conflict and harassment, such as bullying behavior, have a negative influence on peaceful classroom coexistence (Olweus, 1997). Although traditional bullying tends to begin at an early age (Hymel and Swearer, 2015), during adolescence, cyberbullying increases significantly because as age increases, so does the use of mobile phones and the Internet, hence the need to conduct studies focused on this type of bullying (de la Villa Moral and Suárez, 2016).

Several definitions of cyberbullying have been proposed that coincide in their description, pointing out that it is an aggressive and intentional behavior carried out repeatedly by a group of people or an individual, via mobile phones or the Internet without the permission of the victim, who cannot stop these aggressions (e.g., Smith, 2013; Tokunaga, 2010). The aim of aggressors is to intimidate, harass, threaten or harm by sending or posting threatening or humiliating texts, images or videos related to the victim (Slonje et al., 2013). Drawn from these definitions, three key elements of cyberbullying have been identified: (1) power imbalance, (2) multiple repetitions of aggressive behavior, and (3) intention to harm others, (Ybarra et al., 2012). Power imbalance refers to the attempt by the bully to exert control over the targeted victim, who feels helpless or powerless to stop the aggressions. The victim experiences multiple incidents of aggression over a specified time period or feels strongly concerned about it to be repeated. The aggressive behavior is always intentional and meant to produce harm and other negative feelings to the victim (Gladden et al., 2014).

Cyberbullying is a growing problem that exists in all parts of the world without great differences due to cultural, geographical or educational contexts (Zych et al., 2015). According to the UN Educational, Scientific and Cultural Organisation (UNESCO), 2017 report, prevalence data come mainly from industrialized countries and suggest that between 5 and 21% of children and adolescents are affected by cyberbullying worldwide. According to this report, the incidence of cyberbullying increased in Europe from 8 to 12% between 2010 and 2014. In Spain, cyberbullying already accounts for one in four cases of harassment. This proportion increases with age, so that, from the age of 13, 36.5% of cases of harassment (more than one in three) are due to cyberbullying (ANAR Foundation and Mutua Madrileña Foundation, 2017).

The psychological consequences of cyberbullying may be greater than those of traditional bullying due to the lack of space and time constraints and its ability to reach large numbers of people (Li, 2007; Slonje et al., 2013). Thus, adolescents who have experienced cyberbullying report more emotional symptoms and social problems than victims of traditional bullying (Elipe et al., 2015). There is also sufficient empirical evidence on the negative effects of cyberbullying on mental health and psychological adjustment in the long term (Albin, 2012). It appears that the negative consequences of cyberbullying are especially severe during adolescence due to the major neurobiological, cognitive, emotional, and social transformations occurring during this developmental phase (Pabian and Vandebosch, 2016). At this age, changes occur in regions of the brain involved in the processes of emotional regulation, which have important implications for psychological adjustment and social functioning (McRae et al., 2012; Silvers et al., 2017).

Cyberbullying also has a negative effect on subjective well-being, specifically on adolescents’ satisfaction with life (Moore et al., 2012; Navarro et al., 2013). People’s overall assessment of their own lives is considered the cognitive component of subjective well-being, while the affective component refers to positive and negative affects (Diener et al., 2003). Life satisfaction is a key variable in adolescents as an indicator of subjective well-being and optimal social functioning (Proctor et al., 2009). With regard to the school context, students who have been bullies and/or victims of bullying over mobile phones and the Internet report that they are less satisfied with their lives than their peers (29% vs. 40%) (UN Educational, Scientific and Cultural Organisation (UNESCO), 2017).

In summary, school violence endangers peaceful coexistence in the classroom, as well as students’ life satisfaction (Navarro et al., 2013; Tokunaga, 2010). Among the factors that influence bullying are the so-called emotional competencies (e.g., Kokkinos and Kipritsi, 2012; Schokman et al., 2014; Beltrán-Catalán et al., 2018).

### The Role of Emotional Skills and Competencies

Social and emotional competencies seem to influence the development of bullying behavior (Kokkinos and Kipritsi, 2012), but their effect on cyberbullying is still not clear (Beltrán-Catalán et al., 2018). Learning to live together necessarily involves emotional aspects that must be part of the student’s competence to relate to others (Vallés, 2013). According to Mayer et al. (2008, 2016), emotional ability refers to knowing how to identify one’s emotions and feelings and those of others, increase one’s emotional understanding and regulate negative emotions such as anger, fear, and other negative moods (hatred, contempt, animosity, jealousy, etc.) that are often present in classroom conflicts. This emotional learning is an important challenge in the educational context and requires the implementation of an emotional education that complements or is integrated into the contents of education for peaceful school coexistence (Zeidner et al., 2002; López-González and Oriol, 2016).

Emotionally intelligent adolescents tend to be more aware of their own emotions, express their feelings accurately, and regulate emotional responses effectively, thus fostering their emotional and intellectual growth (Mayer et al., 2008). In addition, they demonstrate higher levels of social support and maintain positive and healthy social relationships by meeting the emotional needs of their friends. Furthermore, students with high emotion regulation skills communicate unpleasant moods without offending and manage emotional conflicts and everyday challenges effectively (Brackett et al., 2011). Hence, emotional competencies seem to play a key role in social functioning and peer relationships (Brackett et al., 2006; Peachey et al., 2017), which in turn enhances adolescents’ subjective well-being (Serrano and Andreu, 2016).

Studies that analyze the benefits of developing emotional skills highlight, among other aspects, that they are an important protection factor against the negative consequences of cyberbullying victimization (Baroncelli and Ciucci, 2014), since they could cushion mental health problems (Davis and Humphrey, 2012) by promoting adolescents’ life satisfaction (Geng, 2016). On the other hand, deficits in emotional processing play a crucial role in aggressive behaviors at different levels, so emotional competencies may help to explain the processes involved in peer harassment behaviors (García-Sancho et al., 2017).

Likewise, the positive association between emotional competence and subjective well-being has been investigated over the past decades (Di Fabio and Kenny, 2016; Sánchez-Álvareza et al., 2016; Szczygieł and Mikolajczak, 2017). In general, people with high skills in perceiving, expressing, understanding, and managing emotions resolve emotional conflicts more successfully and therefore be more satisfied with their lives (Mayer et al., 2016). However, the mechanisms that link improvement in peer coexistence and subjective well-being with social-emotional skills in young people have not been studied (Extremera et al., 2018). However, the role of emotional competence as a buffer against the negative impact of cyberbullying on adolescents’ life satisfaction seems to be a promising research approach.

In summary, emotional education in the school population could act as a protective factor against the development of bullying behavior and as a buffer against the negative psychological repercussions of such behavior (Espelage et al., 2015; Sánchez-Calleja et al., 2016). This requires the design of effective, scientifically based interventions to develop students’ emotional abilities that are relevant to addressing the negative consequences of bullying on victims, offenders and all those involved (Garaigordobil et al., 2016).

Evidence-based approaches for teaching social and emotional skills in schools, also known as social and emotional learning (SEL), has gained strength over the last 20 years, and in some countries, policies have been incorporated to promote its integration into the classroom (Torrente et al., 2016). SEL enhances the emotional knowledge and abilities of the whole school community, including children and adults, across all grade and school levels. The approach involves the idea that incorporating social learning and emotional competencies into the core academic curriculum and providing training and support for all school members would promote school coexistence and students’ well-being (Torrente et al., 2016; Taylor et al., 2017).

For instance, most SEL programs have been shown to be effective in improving social-emotional skills in children and adolescents (e.g., Espelage and Hong, 2017; Torrente et al., 2016; Taylor et al., 2017). In addition, these programs have shown positive results on psychological adjustment (Sklad et al., 2012), coexistence and a supportive environment in the classroom (Rivers et al., 2013), self-esteem and self-control (Coelho et al., 2016), and student well-being (Taylor et al., 2017). In addition, some interventions have effectively prevented emotional symptoms such as depression and anxiety (Durlak et al., 2011), conflict in the classroom (Machado Azeredo et al., 2015), sexual harassment perpetration (Espelage et al., 2015), and aggressive behavior (Castillo-Gualda et al., 2018).

With regard to the prevention of school violence, programs are geared toward interventions on cyberbullying (Della Cioppa et al., 2015; Del Rey et al., 2018), and it has been shown that social-emotional development during adolescence may have a positive impact on the classroom climate and school coexistence (Garaigordobil et al., 2016) and is negatively associated with school violence (Peachey et al., 2017). In light of the effectiveness of school interventions in general, the school environment is the ideal place to foster social-emotional development during adolescence.

Drawing from the presented literature on emotional education programs, we developed a novel social and emotional-skill intervention for adolescent population: PREDEMA. The theory underlying PREDEMA is the ability model of emotional intelligence (Mayer and Salovey, 1997) and the dialogical paradigm oriented to meaningful learning (Flecha, 2000). Hence, the purpose of the program was that students learn and apply the skills of emotional abilities through dialogue between the teacher and the student, as well as between the adolescent himself and his emotional reality, allowing them to find meaning in his learning experience (Racionero and Padrós, 2010). To our best knowledge, there has been no study on an evidence-based emotional education program that also monitor changes in school coexistence in terms of cyberbullying and the subjective well-being of adolescents.

### Present Study

Research on emotional education interventions in the school setting confirms that social-emotional competencies can significantly influence young people’s successful development, well-being and optimal social functioning (Torrente et al., 2016). However, despite this evidence, research on the effectiveness of the programs during the months following the intervention is scarce (Weissberg et al., 2015). Moreover, despite the extensive literature supporting the relationship between emotional competence and peer coexistence on the one hand (e.g., Baroncelli and Ciucci, 2014), and subjective well-being on the other (e.g., Peachey et al., 2017), there is still insufficient data to understand how these constructs are related to each other during adolescence.

For all these reasons, this study aims to fill an important gap in the literature on early middle adolescence. A program of social-emotional intervention PREDEMA was designed that is capable of enhancing not only peer coexistence but also adolescents’ well-being. The contribution of this study lies in determining the sequence of the mechanisms involved using path analysis, helping us to understand the process of change. The analysis of the change process is presented both at the end of the program and after 6 months of follow-up. We hypothesized that our social-emotional intervention (1) would develop and improve participants’ emotional skills (perceiving, understanding and regulating emotions); (2) would significantly enhance cognitive aspects of the participants’ subjective well-being, specifically life satisfaction; and (3) would decrease the incidence of cyberbullying through the participants’ learning of peaceful coexistence in the classroom.

## Materials and Methods

### Participants

For this study, a convenience sample of 360 adolescents was chosen, with the following inclusion criteria: (1) interest of their school in participating in the research project, (2) availability to collaborate in the evaluation and intervention during a whole school year, and (3) no previous participation in any SEL programs in the school. The preselected students were randomly divided into two groups: the experimental group, composed of 168 participants, and the control group, composed of 192 adolescents who participated in an alternative intervention proposed by the school. Data were collected in three waves: before the intervention program (preintervention, T1), immediately after intervention was completed (postintervention, T2), and at 6 months follow-up (follow-up, T3). Of the initial 360 participants who responded to the first evaluation (preintervention), data from 28% of the participants was lost in the postintervention period and 43% in the follow-up.

The final sample of this study comprised 148 adolescents (64 boys and 84 girls) aged 12–15 years (*M* = 12.63, *SD* = 0.74). Participants were in their first and second year of compulsory secondary school from four different high schools in the Valencian Community: 88 were in 7th grade and 60 were in 8th grade; 73 attended private schools and 75 attended public schools. Participating schools were similar in size and number of students, as well as ethnic and socio-economic background.

### Study Design and Data Collection

For this study, a quasi-experimental design was used with an intervention (experimental) and a control group. Allocation of schools to the experimental or control group was carried out at random. *T*-tests of independent samples and Chi-squared tests were performed prior to the intervention, indicating that the intervention (*n* = 72) and control group (*n* = 76) did not differ significantly (*p* ≥ 0.05) in any of the studied variables, indicating a correct random allocation.

The research team contacted the schools that had indicated their intention to participate in the project. Information sessions for parents and schoolteachers were organized to explain the nature of the research and present the objective of the intervention program. All students participated in the study voluntarily, with the prior consent of their parents or legal guardians. As mentioned before, data were collected in three waves (T1, T2, and T3) by means of self-reports and after signing an informed consent form (full dataset for this study is included in the Supplementary Material). The control group carried out the three evaluations under the same conditions as the experimental group: as a group, in the classroom, during school hours, with a duration of 50 min. Students from the control group, who didn’t participated in the intervention program, had access to regular resources and cyberbullying prevention protocols provided by the schools, for instance, school counseling or peer mediation programs.

### Ethics

The data were collected according to the standards of the Declaration of Helsinki (World Medical Association, 2013), with permission from the Department of Education, Culture and Sport of the Valencian Community and the Ethics Commission of the University of Valencia (H1385330676977). The results of the study are presented following the indications of APA for quantitative research in psychology (Appelbaum et al., 2018).

### Measurements

The measurements used in this study have adequate psychometric properties of reliability and validity, and they have been adapted and validated in Spanish samples. The Cronbach’s alpha indices reported here correspond to the sample of this study.

#### Emotional Competencies

Emotional competencies were evaluated through the Emotional Skills and Competencies Questionnaire (ESCQ; Takšić et al., 2009; adapted to Spanish by Extremera and Fernández-Berrocal (Faria et al., 2006). It consists of 45 items with six response alternatives (1 = Never; 6 = Always). The instrument evaluates three factors: emotional perception and understanding (α = 0.90); emotional expression and labeling (α = 0.88); emotional management and regulation (α = 0.85). The reliability of the subscales has been proven in previous studies (α = 0.74–0.86) (Faria et al., 2006).

#### Cyberbullying

Cyberbullying was estimated using two different scales. On the one hand, the cybervictimization dimension was evaluated through the victimization scale via mobile phone and internet (CYB-VIC; Buelga et al., 2012). This one-dimensional scale consists of 10 items with five response values (1 = Never; 5 = Many times). The cybervictimization scale presents good internal consistency in this study (α = 0.82) and previous studies (α = 0.84) (Buelga et al., 2012). On the other hand, the incidence of cyberaggression was evaluated using the cyber-aggression scale using mobile phones and the internet (CYB-AG; Buelga and Pons, 2012). The scale provides a general incidence of bullying behavior using mobile phones or the Internet to harass and mock. It is composed of 10 items (α = 0.68) that are rated on the four-point Likert scale (1 = Never; 4 = Many times). Its validity and reliability have also been confirmed in previous studies; Cronbach’s alpha ranges from 0.88 to 0.89 (Buelga and Pons, 2012).

#### Subjective Well-Being

Subjective well-being was assessed using the Satisfaction With Life Scale (SWLS; Diener et al., 1985; validated by Atienza et al., 2000). This scale consists of five items with five response values (1 = strongly disagree, 5 = strongly agree). The validity and internal consistency in the present study was suitable (α = 0.81), as has been confirmed by previous studies (α from 0.79 to 0.89) (Diener et al., 1985).

### Intervention Program: PREDEMA

Guided by Mayer and Salovey (1997) emotional intelligence model and the theory of dialogical learning (Flecha, 2000), PREDEMA was designed to promote classroom coexistence and subjective well-being by developing emotional competencies in adolescents. The program was implemented in six classes with 25–30 students each by a trained psychologist. It consisted of eleven sessions, each of 50 min, which took place over a 3-month period of tutoring time. The first part of the program (sessions 1–6) focused on the most basic emotional abilities, including perceiving, labeling, expressing, using and understanding emotions. The second part (sessions 7–11) targeted emotional regulation and management in different contexts and situations. In addition, complementary issues were discussed, such as personal and global values, responsibility and tolerance, as well as preventing interpersonal conflicts. The sessions started with exploring a personal or emotionally experience, followed by creating a symbolic representation or meanings of the experience in order to integrate it into previous knowledge, and finally transferring the experience to other contexts and discussing the relevance for future experiences. Each week participants were given home practice activities and a worksheet in which to record their daily experience during the week. For further description of the different activities, contents and procedures of the intervention program, see Montoya-Castilla et al. (2016).

### Statistical Data Analysis and Sample Size

Before testing the hypothesized models, descriptive analyses, Pearson correlations and multivariate and univariate variance analysis (MANOVA and ANOVA) were performed to identify possible differences between the experimental group and the control group at baseline (T1). In addition, multivariate and univariate covariance analyses (MANCOVA and ANCOVA) were performed to identify changes in postintervention (T2) and follow-up (T3), controlling for preintervention scores (covariable). In addition, the effect size (Cohen’s *d*) of each variable was calculated to estimate the magnitude of the differences between experimental and control groups (Lipsey and Wilson, 2001). These analyses were performed with the statistical package SPSS V.24.

**FIGURE 1:**
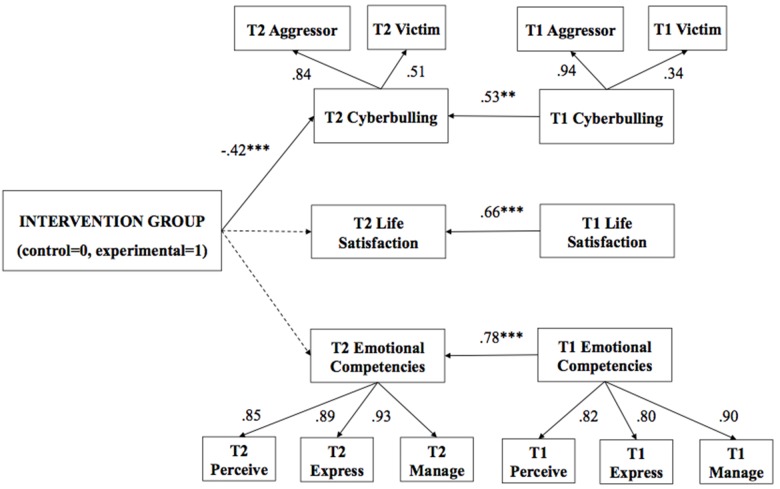
Path analysis at postintervention: The impact of the intervention group on all measured variables (*N* = 148). T1, pre-intervention; T2, postintervention. Bold pathways are significant at *p* < 0.01, dotted pathways are not significant. Factor loadings and estimators (ß) are standardized.

**FIGURE 2:**
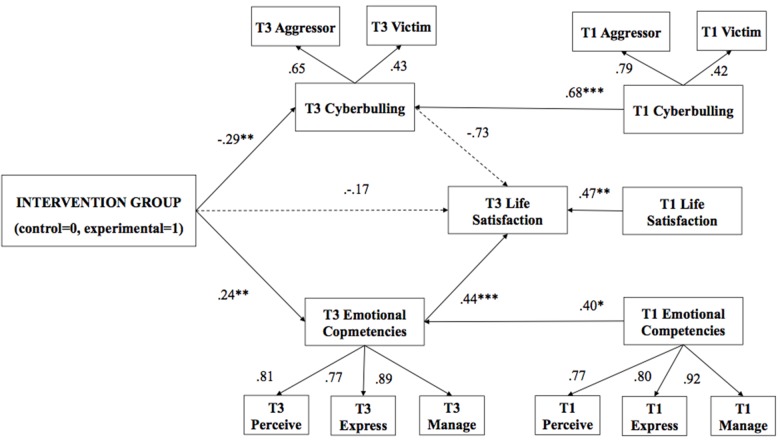
Path analysis at follow-up with mediation: The impact of the intervention group on all measured variables at follow-up (*N* = 148). T1, pre-intervention; T3, follow-up. Bold pathways are significant at *p* < 0.01, dotted pathways are not significant. Factor loadings and estimators (ß) are standardized.

The path analysis was then performed with the final sample (*N* = 148). There is no consensus in literature about an appropriate sample size for conducting structural equation modeling (SEM) (Wang and Wang, 2012). A sample of *N* = 100–150 is usually considered the minimum to test simple SEM models, such as the path models proposed in this study (Tabachnick and Fidell, 2007). In order to examine the impact of the intervention on T2 (Figure 1) and T3 (Figure 2) two different models were tested. The two tested path analysis models were theoretically founded and based on the results of the previous correlation analysis and analysis of variance. Thus, in both models, the intervention group (1 = experimental, 0 = control) was included as a predictor of the variables evaluated in postintervention (T2) and follow-up (T3), controlling the effect of preintervention evaluations (T1). The first model proposes a multiple regression with direct paths from the intervention group to emotional competencies T2, indicated by perception T2, expression T2 and regulation T2; cyberbullying in T2, indicated by cybervictimization T2 and cyberaggression T2; and satisfaction with life T2. The second model proposes an indirect path from the intervention group on satisfaction with life T3 through emotional competencies T3 and cyberbullying T3. In addition, direct paths from T1 variables were included that reflect the initial levels of emotional competence, cyberbullying and life satisfaction in the two models to provide a rigorous test.

The five main indices recommended in the literature were used to evaluate the model fit (Hu and Bentler, 1999): the comparative fit index (CFI), Tucker-Lewis index (TFI), for which a value of 0.90 or higher is usually considered appropriate for accepting the model; the root mean square error of approximation (RMSEA), parsimony index and measure of the amount of error, with values of less than 0.08 considered acceptable to state that a model is plausible; the standardized root mean square residuals (SRMR), as an absolute index that shares criteria with the previous one, and the Robust Chi-Square Test of Model Fit χ^2^ with degrees of freedom (*df*) (Kaplan, 2000; Kline, 2016). Path analyses were performed using Mplus 7.0 with *MLR* (maximum likelihood estimation with robust standard errors) for non-normal data (Muthén and Muthén, 2017).

## Results

### Preliminary Results

The results of MANOVA with baseline scores (Table 1) indicated that there were no differences between the intervention and the control group in T1 (Wilks’ lambda, λ = 0.964, *F*(104) = 0.651, *p* = 0.689, η = 0.036). No significant differences were observed between the experimental and control groups in any studied variables, which indicates a high level of homogeneity between the experimental and control groups.

**Table 1:** Means, SDs, effect sizes, analysis of variance, and analysis of covariance.

		Experimental group	Control group	Cohen’s *d* [95% CI]	ANOVA		ANCOVA	
		*M (SD)*	*M (SD)*		*F*	*p*	*F*	*p*
Perceive emotions	T1	67.00 (11.97)	68.08 (11.53)	0.09 [−2.10, 1.91]	0.01	0.94		
	T2	65.78 (13.84)	68.29 (13.45)	−0.19 [−2.51, 2.14]			1.91	0.17
	T3	70.57 (9.80)	65.17 (12.35)	0.49 [−1.41, 2.39]			15.60	<0.001
Express emotions	T1	58.41 (11.97)	61.70 (11.85)	−0.28 [−2.31, 1.75]	0.83	0.36		
	T2	57.17 (11.97)	61.29 (12.16)	−0.34 [−2.40, 1.71]			1.58	0.21
	T3	58.63 (11.86)	57.57 (12.05)	0.09 [−1.95, 2.13]			0.81	0.37
Manage emotions	T1	73.84 (11.11)	75.08 (11.54)	−0.11 [−2.04, 1.82]	0.04	0.84		
	T2	74.03 (11.97)	75.61 (12.55)	−0.13 [−2.22, 1.96]			0.45	0.50
	T3	76.70 (8.26)	70.97 (10.57)	0.61 [−1.01, 2.23]			16.41	<0.001
Cyberaggression	T1	12.78 (3.13)	12.36 (2.77)	0.14 [−0.36, 0.65]	0.23	0.63		
	T2	11.46 (2.24)	13.96 (5.46)	−0.60 [−1.31, 0.11]			23.27	<0.001
	T3	11.42 (2.05)	12.82 (4.86)	−0.38 [−1.01, 0.26]			6.86	0.01
Cybervictimization	T1	12.78 (2.97)	12.82 (3.09)	−0.01 [−0.53, 0.50]	0.47	0.50		
	T2	11.88 (2.45)	13.92 (5.02)	−0.52 [−1.19, 0.15]			14.42	<0.001
	T3	12.35 (2.62)	13.01 (3.25)	−0.23 [−0.73, 0.28]			1.19	0.28
Life satisfaction	T1	26.38 (6.00)	27.36 (5.27)	−0.18 [−1.13, 0.79]	0.59	0.44		
	T2	27.46 (5.78)	28.75 (5.51)	−0.23 [−1.19, 0.73]			0.84	0.36
	T3	27.63 (5.66)	26.27 (6.63)	0.22 [−0.83, 1.27]			5.86	0.02

*M, mean; SD, standard derivation; Cohen’s d, effect size; CI, confidence interval; F, F ratio; p, probability; T1, pre-intervention; T2, postintervention; T3, follow-up.*

With regard to T2 and T3 (Table 1), significant differences were observed between the experimental condition and short-term control (Wilks’ lambda, λ = 0.81, *F*(6) = 2.79, *p* = 0.017, η = 0.195). Specifically, after participating in the intervention program, the experimental group improved significantly in cyberaggression and cybervictimization in T2 compared to the control group. The effect size was moderate-high in both cases. These immediate changes were maintained over 6 months (Wilks’ lambda, λ = 0.746, *F*(6) = 4.77, *p* = < 0.001, η = 0.254). Thus, differences were observed between the experimental group and the long-term control group in emotional perception, emotional regulation, cyberaggression, and satisfaction with life in T3, with a moderate to large effect size.

### Correlations

Pearson correlations were performed for all studied variables (Table 2). The results indicated that variables in T1 were significantly correlated with the corresponding measure at T2 and T3. In addition, significant positive correlations were observed between emotional competencies T2 and life satisfaction T2, as well as between emotional regulation T3 and life satisfaction T3. Also noteworthy is the negative and significant relationship between cyber aggression and life satisfaction in both T2 and T3. Finally, emotional competencies T2 are negatively and significantly related to cyberaggression T2.

**Table 2:** Correlations between study variables (*N* = 148).

	*M*	*SD*	1	2	3	4	5	6	7	8	9	10	11	12	13	14	15	16	17
1. T1 Cyberaggression	12.56	2.95	–																
2. T1 Cybervictimization	12.80	3.02	0.32^∗∗^	–															
3. T1 Perceive emotions	67.52	11.73	−0.22^∗^	0.04	–														
4. T1 Express emotions	60.11	11.98	−0.15	−0.03	0.66^∗∗^	–													
5. T1 Manage emotions	74.43	11.29	−0.29^∗∗^	−0.05	0.74^∗∗^	0.7^∗∗^	–												
6. T1 Life Satisfaction	26.88	5.64	−0.26^∗∗^	−0.13	0.27^∗∗^	0.45^∗∗^	0.50^∗∗^	–											
7. T2 Cyberaggression	12.74	4.38	0.50^∗∗^	0.21^∗^	−0.13	−0.10	−0.24^∗∗^	−0.26^∗∗^	–										
8. T2 Cybervictimization	12.93	4.10	0.24^∗∗^	0.44^∗∗^	0.01	0.01	−0.13	−0.08	0.44^∗∗^	–									
9. T2 Perceive emotions	67.10	13.64	−0.18^∗^	0.03	0.77^∗∗^	0.63^∗∗^	0.70^∗∗^	0.34^∗∗^	−0.13	−0.04	–								
10. T2 Express emotions	59.42	12.20	−0.13	−0.01	0.57^∗∗^	0.66^∗∗^	0.61^∗∗^	0.47^∗∗^	−0.14	−0.04	0.77^∗∗^	–							
11. T2 Manage emotions	74.83	12.73	−0.15	−0.09	0.57^∗∗^	0.62^∗∗^	0.71^∗∗^	0.46^∗∗^	−0.20^∗^	−0.10	0.81^∗∗^	0.82^∗∗^	–						
12. T2 Life Satisfaction	28.12	5.66	−0.20^∗^	−0.07	0.16	0.30^∗∗^	0.35^∗∗^	0.67^∗∗^	−0.23^∗∗^	−0.01	0.34^∗∗^	0.47^∗∗^	0.47^∗∗^	–					
13. T3 Cyberaggression	12.13	3.80	0.29^∗∗^	0.22^∗∗^	−0.06	0.05	0.02	−0.07	0.25^∗∗^	0.08	0.04	0.06	−0.03	−0.01	–				
14. T3 Cybervictimization	12.68	2.96	0.24^∗∗^	0.44^∗∗^	0.15	0.068	0.04	−0.16^∗^	0.24^∗∗^	0.36^∗∗^	0.17	−0.01	0.04	−0.06	0.30^∗∗^	–			
15. T3 Perceive emotions	67.85	11.44	−0.21^∗^	−0.10	0.62^∗∗^	0.38^∗∗^	0.45^∗∗^	0.17^∗^	−0.11	−0.19^∗^	0.55^∗∗^	0.36^∗∗^	0.38^∗∗^	0.22^∗∗^	0.01	0.06	–		
16. T3 Express emotions	58.08	11.93	−0.11	−0.10	0.32^∗∗^	0.49^∗∗^	0.34^∗∗^	0.29^∗∗^	−0.01	−0.00	0.40^∗∗^	0.51^∗∗^	0.42^∗∗^	0.35^∗∗^	0.00	−0.06	0.60^∗∗^	–	
17. T3 Manage emotions	73.73	9.92	−0.19^∗^	−0.18^∗^	0.32^∗∗^	0.30^∗∗^	0.37^∗∗^	0.26^∗∗^	−0.22^∗^	−0.08	0.43^∗∗^	0.25^∗∗^	0.45^∗∗^	0.32^∗∗^	−0.13	−0.09	0.65^∗∗^	0.63^∗∗^	–
18. T3 Life Satisfaction	0.09	5.36	0.11	0.03	−0.09	−0.20^∗^	−0.198^∗^	−0.37^∗∗^	−0.07	−0.05	0.02	−0.1	−0.07	0.01	−0.28^∗∗^	−0.09	0.14	0.07	0.30^∗∗^

*M, mean; SD, standard derivation; T1, pre-intervention; T2, postintervention; T3, follow-up. ^∗^p < 0.05; ^∗∗^p < 0.01.*

### Path Analyses

The first model (Figure 1), which represents the postintervention change process (T2), showed an satisfactory model fit, except for SRMR: χ^2^: 87.98 (*df*: 46); *p* < 0.05; CFI: 0.95; TLI: 0.91; and RMSEA: 0.07 [0.05–0.10]. The SRMR (0.13) might be positively biased due to small sample size and low *df* (Hooper et al., 2008). In relation to T2 change processes, the intervention group predicted low levels of T2 cyberbullying, while paths to T2 emotional intelligence and T2 life satisfaction were not significant. With respect to the control variables, in the prediction of emotional intelligence T2, cyberbullying T2 and life satisfaction T2 affect the initial T1 levels of the corresponding variables (Table 3). In summary, participants in the emotional education program scored lower on cyberbullying than the control group immediately after the intervention program had finished, controlling for the effect of baseline levels on all variables. However, the changes at T2 in emotional competences and life satisfaction were not statistically significant. The model accounted for 48% of the variance of cyberbullying.

**Table 3:** Path coefficients and model-fit indices for hypothesized mediation models (*N* = 148).

	Model 1: Path analysis T2		Model 2: Path analysis T3		Model 3: Mediation T3	
	ß	95% CI	ß	95% CI	ß	95% CI
**Direct effects**						
Intervention → Cyberbullying	−0.42^∗∗∗^	[−0.59 to −0.25]	−0.29^∗∗^	[−0.51 to −0.07]	−0.29^∗∗^	[−0.49 to −0.09]
Intervention→ Emotional Competencies	−0.05	[−0.17 to 0.07]	0.31^∗∗∗^	[0.19 to 0.43]	0.24^∗∗^	[0.07 to 0.40]
Intervention →Life satisfaction	−0.06	[−0.18 to 0.06]	0.16^∗∗^	[0.04 to 0.28]	−0.17	[−0.51 to 0.16]
Cyberbullying → Life satisfaction	–	–	–	–	−0.73	[−1.58 to 0.12]
Emotional Intelligence → Life satisfaction	–	–	–	–	0.44^∗∗∗^	[0.24 to 0.64]
**Indirect effects**						
Intervention → Cyberbullying → Life satisfaction	–	–	–	–	0.21	[−0.13 to 0.56]
Intervention → Emotional Competencies → Life satisfaction	–	–	–	–	0.10^∗∗^	[0.02 to 0.19]
**Model-fit indices**						
χ^2^(df)	87.98 (46)		84.61 (47)		77.25 (47)	
Δχ^2^(df)	1.87		1.80		1.64	
CFI	0.95		0.94		0.95	
TFI	0.91		0.90		0.91	
RMSEA [90% CI]	0.07 [0.05–0.10]		0.07 [0.05–0.09]		0.06 [0.04–0.09]	
SRMR	0.13		0.06		0.06	

*β, standardized path coefficient; CI, confidence interval; χ^2^, Chi-Square Test of Model Fit; df, degrees of freedom; CFI, comparative fit index; TFI, Tucker-Lewis index; RMSEA, root mean square error of approximation; SRMR, standardized root mean square residuals; ^∗^p < 0.05; ^∗∗^p < 0.01; ^∗∗∗^ p < 0.001.*

The second model (Figure 2), which includes the indirect effects of the intervention group on life satisfaction through cyberbullying and emotional competencies in T3, fits the data better than the non-mediation model: χ^2^: 77.25 (*df*: 47); *p* < 0.05; CFI: 0.95; TLI: 0.91; RMSEA: 0.06 [0.04–0.08]; and SRMR: 0.06. With regard to the mediation model, the intervention group predicted low levels of cyberbullying T3 and high levels of emotional competence T3, while the direct effect to life satisfaction T3 was not statistically significant, indicating complete mediation. However, the indirect effect from the group predicted higher levels of life satisfaction T3 mediated by emotional competencies T3, while the indirect effect mediated by T3 cyberbullying was not significant (Table 3). In summary, emotional competence mediates the relationship between the intervention group and life satisfaction over 6 months. In other words, participants in the emotional education program with better emotional competencies scored higher in life satisfaction than the control group at follow-up, controlling for the effect of the initial levels of both variables. However, the changes in cyberbullying were not related to the changes in life satisfaction. The mediation model explains 52% of the variance in cyberbullying and 32% of the variance in emotional competence. With respect to indirect effects, the total effect represented 14% of the variance of life satisfaction.

## Discussion

Different intervention programs have been designed and implemented to prevent school violence, both traditional (bullying) and via mobile phones and the Internet (cyberbullying), as well as conflicts between peers in the classroom, as they endanger the successful intellectual and social development of students (Garaigordobil et al., 2016; Del Rey et al., 2018). Of the programs for adolescents that focus on the social-emotional competencies proposed in the literature, few have evaluated their effectiveness, focusing on cyberbullying behaviors, in follow-up evaluations (Castillo-Gualda et al., 2018). Taking into account these gaps in the literature on school interventions, the objectives of the present study were to implement an emotional education program and to evaluate its immediate and follow-up effectiveness in promoting peer coexistence, assessed through cyberbullying behaviors, and subjective well-being. It was also intended to identify the process by which the development of emotional skills promotes peer coexistence and subjective well-being during adolescence.

In relation to the first hypothesis, the program was not effective in developing emotional competencies at the follow-up in the intervention group compared to the control group. The intervention group significantly improved their ability to perceive, understand and regulate emotions, although this result was not pronounced immediately after the intervention, only at follow-up. This may be because the knowledge and experiences gained during the program did not have an immediate impact on adolescents. It seems that the social-emotional skills need to be established and matured over time, put into practice in everyday life and thus integrated into one’s own repertoire. These results are in line with the literature that indicates that interventions in the school setting can be effective in developing emotional competencies and strategies in adolescents to better perceive, express, and regulate emotional responses (Coelho et al., 2016; Sánchez-Calleja et al., 2016).

In addition, the results support the second hypothesis that the intervention program would be effective in promoting coexistence in the classroom by reducing the incidence of cyberbullying longitudinally. In this sense, the adolescents who participated in the program reported fewer threatening and humiliating behaviors via mobile phone and the Internet compared to the control group, both immediately after the program ended and after some time had passed. Thus, the emotional education program PREDEMA proved to be effective as a short-term and long-term cyberbullying prevention program in a population as vulnerable as the adolescent. The benefits of social-emotional interventions on bullying have been shown in previous studies (e.g., Tokunaga, 2010; Garaigordobil et al., 2016; Espelage and Hong, 2017; Del Rey et al., 2018). These programs have used different theoretical approaches and methodologies when designing effective interventions to reduce both mobile phone and Internet aggressions as well as cybervictimization in the classroom.

With regard to the third scenario, the intervention program promoted long-term subjective well-being, specifically satisfaction with life for adolescents. Our results indicated a significant improvement in the assessment of the positive aspects they have in their lives when appreciating their own competencies at different levels compared to the control group. These benefits were evident a few months after the intervention. One possible explanation could be the impact that one’s own emotional competencies have on well-being, as both show their effect over time (Sánchez-Álvareza et al., 2016). In particular, emotional competencies mediated the effect of social-emotional intervention on life satisfaction. That is, as soon as adolescents were able to integrate their new knowledge and emotional competencies into their lives, their satisfaction with general aspects of their lives also increased, appreciating the new resources and strategies they had acquired to become better involved in the classroom and maintain positive relationships with peers (Geng, 2016).

In our opinion, this study is a pioneering one that examines the mechanisms that explain the benefits of an emotional education program on the well-being of participants using longitudinal postintervention and follow-up data. In this case, the protective factors of peer coexistence and psychological well-being are examined by considering the processes that may explain their effectiveness (Durlak et al., 2011). In addition, our results provide evidence of the benefits of developing emotional competencies in adolescents, a particularly key evolutionary stage for future personal and intellectual growth. A better understanding of the predictors and mechanisms of subjective well-being in adolescence is very relevant because the mere prevention of problematic and aggressive behaviors does not necessarily imply the presence of mental health and psychological adjustment in adolescents (Davis and Humphrey, 2012).

Our results support the revised model of emotional intelligence (Mayer et al., 2016) that considers emotional intelligence as the ability to solve interpersonal problems by recognizing the emotional needs of peers, understanding the meaning of emotions and their implications for the behavior of others, managing one’s own emotions and those of others to achieve desired emotional states in oneself and in those around one. In this sense, adolescents are more likely to resort to aggressive behavior when they are unable to regulate their unpleasant emotional states and resolve interpersonal conflicts (García-Sancho et al., 2017). According to this theory, people with high levels of emotional competence have greater social support by maintaining strong and healthy bonds with their friends, which protects them from being isolated and helpless in the face of sudden and prolonged aggression (Baroncelli and Ciucci, 2014; Peachey et al., 2017). In contrast, adolescents with few emotional skills are more likely to be victims of bullying and cyberbullying because they have fewer resources and social-emotional strategies to successfully confront threats and humiliations (Extremera et al., 2018).

In summary, our study has different strengths, including a longitudinal design with three evaluations (pre- and post-intervention and a 6-month follow-up), a quasi-experimental design with an experimental and control group, random assignment of classrooms to experimental conditions and rigorous analysis of results using path analysis.

### Limitations and Future Research

Although the results of this study are promising, they must be interpreted in light of some limitations, which might affect external and internal validity of our findings. First, all data used in the study were self-reported, implying a bias of social desirability and increasing the likelihood that relationships would inflate due to the variance of the shared method (Brackett et al., 2006). Future studies should also include other forms of evaluation of cyberbullying, as well as reports from teachers, which will provide a more reliable and comprehensive picture of indicators of coexistence among adolescents. Second, this study was conducted with a sample of Spanish youth, the results may differ from adolescents in other countries with different school systems and/or cultural contexts. Therefore, it would be advisable for future lines of research to use a cross-cultural design to replicate the results, as well as to apply and validate the intervention program with participants from multiple countries. Third, the sample might be biased and only partially representative for the broader population of Spanish adolescent, due to sampling procedures of convenience and dropout effects, decreasing sample size. Finally, in order to improve external validity, future research should consider using a more systematic sampling plan and take actions to avoid participants to abandon the study early.

#### Conclusions and Practical Implications

Despite these limitations, this study makes an important contribution to the literature on interventions in emotional competence with longitudinal data. Our findings indicate that the emotional education program has important implications from two perspectives: promoting peer coexistence in the classroom by reducing cyberbullying and improving the subjective well-being of adolescents in a school setting. Considering that emotional skills and abilities are a predictor of good academic performance, coping strategies, and well-being (Brackett et al., 2011; Geng, 2016; Sánchez-Álvareza et al., 2016), the emotional education program may be a valuable intervention for adolescent mental health at this critical developmental stage (McRae et al., 2012).

These findings will help design programs to prevent bullying in the school environment, supporting psychosocial functioning and the well-being of adolescents. In addition, our results provide a better understanding of the processes involved in the effects of adolescent interventions. In light of these findings, emotional education intervention and prevention programs are appropriate and acceptable for adolescents and should be included in school education plans to increase student self-efficacy and facilitate academic success (Taylor et al., 2017).

The same data can be used by teachers and school counselors to identify adolescents’ social and emotional strengths, their interests and concerns when considering implementing a SEL program such as PREDEMA. To increase the likelihood of successful implementation, future studies should determine whether the program is more or less effective for students with high-risk, identify optimal conditions (location, timing, number of participants) for the intervention, generate a climate of respect among students and make adjustments without harming the integrity of the program (Torrente et al., 2016). Drawn by our own experience and in accordance with others (Lavy and Eshet, 2018), we suggest that educators might consider to engage in a previous training in social-emotional skills themselves. They could then promote these competences in their students in the classroom acting as a socio-emotional role model., before teaching students about these skills.

This would allow progress to be made in understanding the impact of the development of emotional competencies at the inter- and intra-personal levels compared to previous studies with short-term interventions and cross-sectional data (e.g., Garaigordobil et al., 2016; Del Rey et al., 2018). In addition, this study makes an important contribution to the existing literature that supports the role of emotional competencies in peer-to-peer coexistence by preventing the prevalence of cyberbullying behaviors and promoting satisfaction with life among adolescents (Di Fabio and Kenny, 2016; Torrente et al., 2016; Peachey et al., 2017; Castillo-Gualda et al., 2018).

## Author Contributions

All authors participated and contributed in the study design, the data collection, the statistical analysis, the interpretation of data, and drafted the manuscript. Besides, all authors read and approved the final manuscript.

## Conflict of Interest Statement

The authors declare that the research was conducted in the absence of any commercial or financial relationships that could be construed as a potential conflict of interest.
